# “Sticky invasion” – the physical properties of *Plantago lanceolata* L. seed mucilage

**DOI:** 10.3762/bjnano.7.183

**Published:** 2016-12-05

**Authors:** Agnieszka Kreitschitz, Alexander Kovalev, Stanislav N Gorb

**Affiliations:** 1 Department of Plant Developmental Biology, Institute of Experimental Biology, University of Wrocław, ul. Kanonia 6/8, 50-328 Wrocław, Poland; 2 Department of Functional Morphology and Biomechanics, University of Kiel, Am Botanischen Garten 9, D-24118 Kiel, Germany

**Keywords:** adhesion, cellulose mucilage, desiccation, friction, *Plantago lanceolata*

## Abstract

The mucilage envelope of seeds has various functions including the provision of different ways for the dispersal of diaspores. Chemical composition and water content of the mucilage yield particular adhesive and frictional properties in the envelope that also influence the dispersal of seeds. To determine the physical properties of *Plantago lanceolata* seed mucilage we studied (1) composition, (2) desiccation, (3) adhesion, and (4) friction properties of the mucilage under different hydration conditions. We revealed the presence of cellulose fibrils in the mucilage, which are responsible for a continuous and even distribution of the mucilaginous layer on the seed surface. The measured values of adhesive and frictional properties differed significantly in comparison to the previously studied pectic mucilage of *Linum usitatissimum*. Also, the water loss from the cellulose mucilage was more rapid. The obtained different values can result from the presence of cellulose fibrils and their interaction with pectins in the mucilage. Because of this feature the mucilage of *P. lanceolata* may represent a more regularly ordered and stabile system than the pectic mucilage of flax, which lacks cellulose. In spite of the fact that *P. lanceolata* mucilage revealed different adhesive and frictional properties than the pectic mucilage, it still demonstrates an effective system promoting zoochoric seed dispersal. Cellulose may additionally prevent the mucilage against loss from the seed surface.

## Introduction

The ability of seeds and fruits (diaspores) to form mucilage after hydration is known as myxospermy [1–2]. The mucilaginous diaspores are particularly characteristic of plants that grow in dry or disturbed habitats [1,3]. The presence of mucilage results in different benefits for the plant including (1) fixation of diaspores to the ground, (2) water supply essential for germinating an embryo or (3) egzo- and endozoochoric dispersal by animals [1–3].

*Plantago lanceolata* L. (narrow-leaf plantain) is a perennial herb known as a common weed, widely distributed in grasslands and roadsides of temperate regions of the world. It grows on a wide range of soils and is resistant to drought [4–6]. *P. lanceolata* is spread throughout the whole world excluding subarctic and low-lying tropical areas [5,7]. Species of the genus *Plantago* are used as a source of mucilage produced by vegetative parts [6] as well as by seeds [1].

*Plantago* seeds are dispersed by animals [8–9]. They can be eaten by sparrows and then spread by their droppings (endozoochory). Also, they can stick to the feet of the animals, the fur or the bird plumage enabling further dispersal (epizoochory) [8]. *P. lanceolata* seed mucilage was identified as an acid polysaccharide complex containing, in addition to pectins, different sugars, e.g., pentose, galactose, xylose [10–11]. The mucilage composed mainly of pectins is described as pectic type ("true slime") and is typical of, e.g., *Linaceae, Plantaginaceae* and *Poaceae* seeds [1,12]. Often mucilage possesses an additional cellulose skeleton and is then classified as cellulose mucilage, characteristic of taxa from many diverse families like Asteraceae, Brassicaceae, Lamiaceae [12–15].

Mucilage is produced by a special type of cells, namely mucilage-secreting cells (MSCs), which are an integral part of the seed/fruit coat. The cell wall of these particular cells undergoes modification during seed development, particularly resulting in an increased content of pectins [16]. Apart from pectins, hemicellulose (e.g., arabinoxylan) was also detected in *Plantago* mucilage [16]. Chemical analyses and diverse experimental techniques revealed that the mucilage comprised of a complex composition and structure that can be considered as a specialized pectin-rich secondary cell wall [17].

In the typical cell wall, two coexisting systems are present, one composed of cellulose–hemicellulose and one composed of pectins. They can interact with each other to form the network structure of the cell wall [18–20]. The spatial structure of the cell wall is also maintained through diverse bonds such as covalent bonds and ionic or hydrogen interactions [21]. Consequently, the interaction between individual components of the cell wall can influence its physical characteristics such as its mechanical properties [22].

The physical properties of the cell wall depend on its chemical composition. For example, the addition of certain matrix substances decreases the cell-wall stiffness [23], and lignification causes a higher mechanical strength [24]. Such changes of mechanical properties can also be seen in the case of mucilaginous cell wall. This wall that is rich in pectins form elastic, gel-like mucilage envelope after hydration [14,17]. Other components of the mucilage envelope such as cellulose fibrils also influence its properties and consequently its function.

Seed mucilage possesses a clearly defined nanostructure. Cellulose is a linear homopolymer of 1,4-ß-D-glucan units [25]. Cellulose chains are linked together to form elementary fibril, the size of which can vary depending on the cell-wall type (primary, secondary) and the presence of other components (hemicelluloses) coating the surface. The size of microfibrils, estimated for the primary cell wall, was in a range from 8 to 15 nm up to 30 nm [19,26–27]. Also, the fibrillary material of seed mucilage can vary in its size from thin cellulose fibrils of quince with about 5 nm [28] to fibrillary material of chia (*Salvia hispanica*) ranging between 15 to 45 nm [29]. Cellulose constitutes a kind of scaffold for other mucilage components, namely pectins and hemicelluloses, spread between the fibrils. They represent linear and/or branched polymers with size about 7 nm [27]. Considering the composition and the size of the individual components, mucilage can be treated as a specific kind of nanobiomaterial.

Cellulose is well known as natural and renewable plant polymer. Nano-cellulose is used in biomedicine, cosmetics and food industry [30]. Mucilage, which contains diverse polysaccharides, among them cellulose, exhibits a high cohesive and adhesive properties. Due to this features mucilage finds diverse applications in pharmacy as, e.g., tablet binders, disintegrants, emulsifiers, and suspending or thickening agent. Mucilage as polymer was also studied for the application in pharmaceutical dosage as, e.g., film coating agents, buccal films or nanoparticles [31].

In the study presented here, we examined adhesive and frictional properties of the mucilage of *Plantago lanceolata* and compared them to those previously studied for *Linum usitatissimum* [32]. Cellulose mucilage represents a much more developed and organized system in comparison to the simple pectic mucilage of *Linum usitatissimum* [1]. In addition to the characterization of mucilage, three types of experiments were performed in this study with *P. lanceolata* mucilaginous seeds: (1) measurement of the desiccation dynamics of the hydrated seeds; (2) pull-off force estimation and (3) characterisation of frictional properties. The latter two experiments were performed using different hydration conditions of the mucilage envelope. We used in our experiments the same devices, methods and conditions as in the previous work [32].

The following questions were asked: (1) Is there difference in the dynamics of water loss in different types of the mucilage? (2) How strong is the adhesion of the cellulose-containing mucilage at different stage of its desiccation on the smooth stiff surface? (3) How does the desiccation change frictional properties of the cellulose-containing mucilage? (4) How do these properties differ between the cellulose mucilage type (*Plantago lanceolata*) and previously studied pectic mucilage (*Linum usitatissimum*) [32]? (5) How can the studied physical properties of *P. lanceolata* mucilage influence its seed dispersal?

## Experimental

### Composition of *Plantago lanceolata* mucilage

Mature seeds were collected along a dirt road in the wilderness near Wrocław, Poland. To examine the mucilage composition, staining reactions with 0.1% aqueous solution of ruthenium red (pectin staining), with 0.01% aqueous solution of methylene blue (cellulose staining) and with 0.1% aqueous solution of Direct Red 23 (specific for cellulose) were performed [15,33–35]. Crystalline cellulose in the mucilage envelope was analysed on the hydrated seeds using a polarized-light microscope Leica connected to camera Leica DFC 450 C and Las X software (Leica DM 6000B, Leica Microsystems GmbH, Germany). The images were taken using an Olympus BX-50 light microscope connected to a DP71 camera with Cell B imaging software (Olympus BX50, Olympus Optical Co, Poland) and Zeiss CLSM microscope (LSM 700 AXIO ZEISS, Germany; excitation 555, emission >560 nm)

For further experiments, seeds were hydrated in distilled water for 30 min to obtain the mucilage envelope. In each experiment, new seed samples were used.

For the next three experiments the whole experimental details and procedures have been carried out following the previous studies for *Linum usitatissimum* mucilaginous seeds [33].

### Desiccation dynamics of the mucilage

The seeds of *P. lanceolata* were taken to perform experiments of desiccation dynamics. Five individual seeds were used and for each seed an individual measurement was carried out. After determination of the dry seed mass the seed was hydrated for 30 min to obtain mucilage envelope. Then the dynamic of water loss from the hydrated seed was continuously measured until the initial dry mass was reached. To control the weight measurements an Ultra Microbalance UMX2 and software Balance Link (Mettler-Toledo GmbH, Greifensee, Switzerland) were used.

During the experiments, room temperature and relative air humidity were continuously recorded using a Tinytag TGP-4500 (Gemini Data Loggers Ltd, United Kingdom). The measured temperature was 22–23 °C, the relative humidity was 30–37%.

### Pull-off force measurements of the mucilage

For the pull-off force measurements five sets of measurements were made on five individual seeds. For the measurements a setup [32] consisting of force transducer, motorized micromanipulator (FORT100, World Precision Instruments, Sarasota, FL, USA), data acquisition system and software AcqKnowledge 3.7.0 (Biopac Systems Inc., Goleta, CA, USA), binocular microscope Leica MZ 12-5 (Germany) and video camera KODAK Motion Corder Analyzer Series SR (Eastman Kodak Co., San Diego, CA, USA) was used. The measurements were done on hydrated seeds (with developed mucilage envelope) and were performed until the adhesion force was no longer detectable. Images of the mucilage contact area were taken during the measurements, using a binocular microscope equipped with a video camera (KODAK) with coaxial illumination.

### Friction measurements of the mucilage

A series of five measurements, each based on an individual seed set consisting of five seeds, was made. Five seeds were attached to the tilting platform [32]. After 30 min of seed hydration the measurements were started and continued until the mucilage completely dried out. For the measurements four different loads were used: (1) 15.7 mN, (2) 32 mN, (3) 47.1 mN and (4) 63.8 mN. These loads were applied repeatedly in ascending order (load 1, load 2, load 3, load 4, load 1 and so on). The seeds were fixed to a tiltable platform by adhesive tape and the load was applied in the form of a glass block with corresponding mass lying on the seeds. Then, the platform was tilted until the glass block began to move relative to the seeds. The tilt angle was determined from the built-in alidade and then used for calculating the static friction coefficient. The friction coefficient and adhesion force were both calculated from the measured results as described in [32]. Shortly, the critical angle, φ_c_, at which the glass block started sliding corresponds to the following equation:

[1]
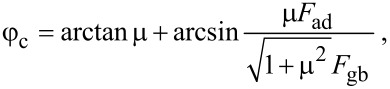


where µ is the friction coefficient, *F*_ad_ is the adhesion force, *F*_gb_ = *m*_gb_·*g*, *m*_gb_ is the mass of the glass block, and *g* = 9.813 m/s^2^ is the gravitational acceleration. At the same time, a special normalization procedure was carried out within each measurement set to obtain a smooth time dependence of the friction coefficient and adhesion force. The penalty function to the least square fit (according to Equation 2) was introduced, which is a second derivative of the estimated parameters with respect to time [36]. The friction coefficients and adhesion forces were found for each time using the following equation:

[2]
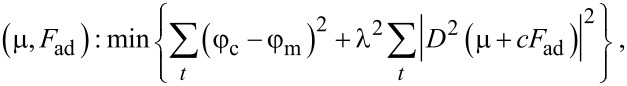


where the first sum minimizes the deviation of the estimated slip angle from the measured slip angle and the second sum is a regulatory term smoothing the curves of μ and *F*_ad_. *D^2^* is an operator of the second derivative of time, λ is a regulatory parameter, *c* is a weighing factor between friction coefficient and adhesion force contributions. The regulatory parameter was selected to dispose of high amplitude peaks in the time dependence of the friction coefficient and to reproduce the shape of the time dependence of the adhesion force. Calculations were performed in Matlab 7.10 (The MathWorks, Natick, NA, USA).

### Statistical analysis

Statistical analyses were performed with OriginPro 8 (OriginLab Corporation, Northampton, MA, USA). Comparison of means was performed with a two-sample t-test. Normal distribution and constant variance of data were verified before statistical analysis, and the *P*-value was set to *P* < 0.001 if one of both conditions was not achieved.

## Results

### Mucilage composition

**Figure 1:**
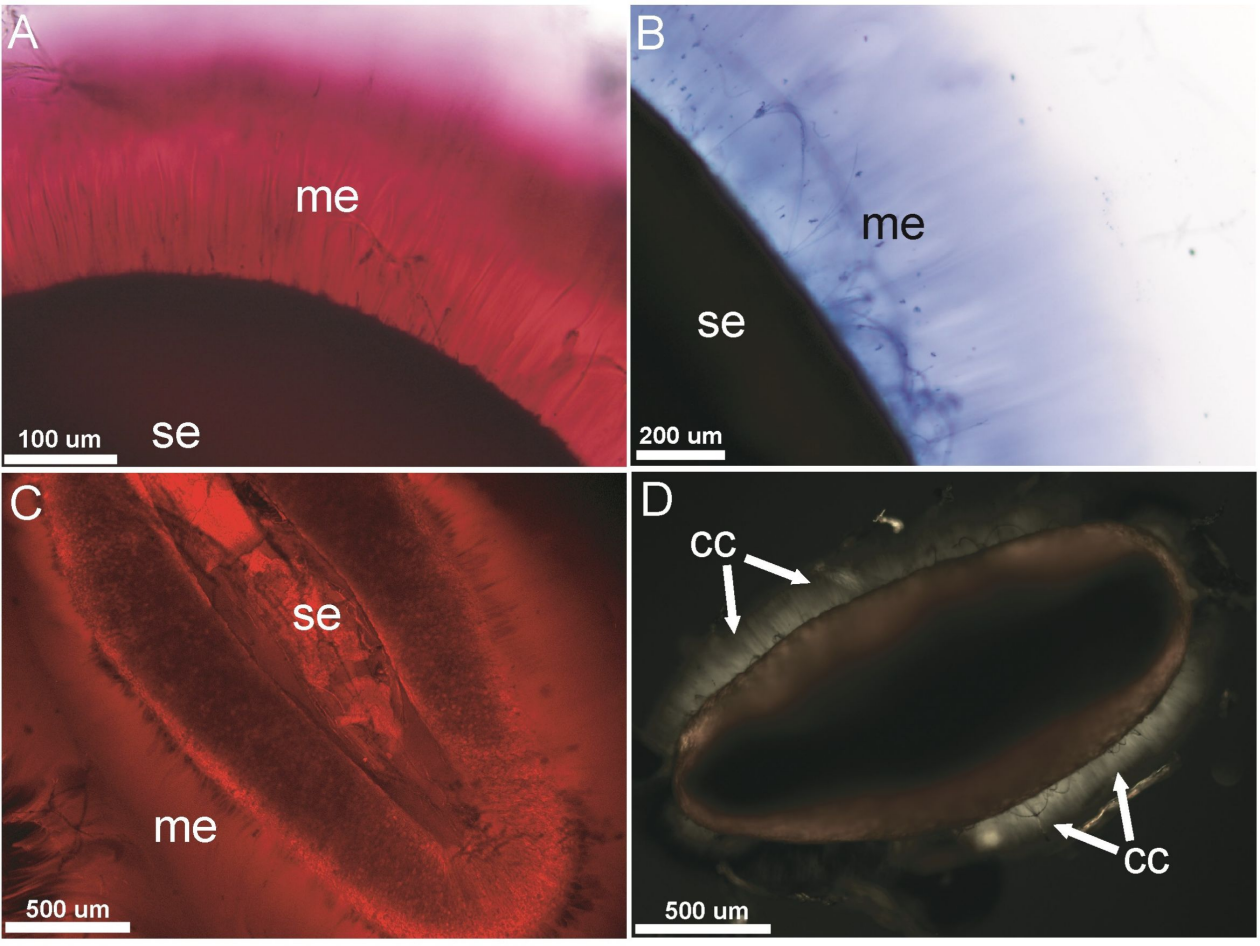
Mucilage composition. Bright field and fluorescence microscopy images of different staining reactions: A – pectin staining with ruthenium red; B – cellulose staining with methylene blue; C – cellulose staining with Direct Red 23, fluorescence image; D – detection of crystalline cellulose in polarized light. Characteristic signal of the cellulose fibrils (arrows); Cellulose fibrils are imbedded in the mass of pectins (cc – crystalline cellulose, me - mucilage envelope, se - seed).

The mucilage of *Plantago lanceolata* contained both pectin and cellulose components, which are characteristic of cellulose mucilage. Positive staining with ruthenium red (Figure 1A) revealed the presence of pectins in the mucilage. Staining with methylene blue (Figure 1B) and Direct Red 23 (Figure 1C) revealed the presence of cellulose. The cellulose fibrils kept the mass of pectins in place. The thickness of the mucilage envelope ranged from 350 to 450 μm. Cellulose fibrils were very delicate and embedded in the voluminous pectin mass. Crystalline cellulose in the mucilage was also visualized using a polarized light microscope (Figure 1 D). After hydration, the fibrils build a regular, radial “skeleton” (Figure 1 D) and, together with pectins, form a mucilage envelope surrounding the seed. Characteristic feature of *P. lanceolata* seed was that the mucilage envelope formed a continuous and evenly distributed layer on the seed surface.

### Desiccation dynamics

**Figure 2:**
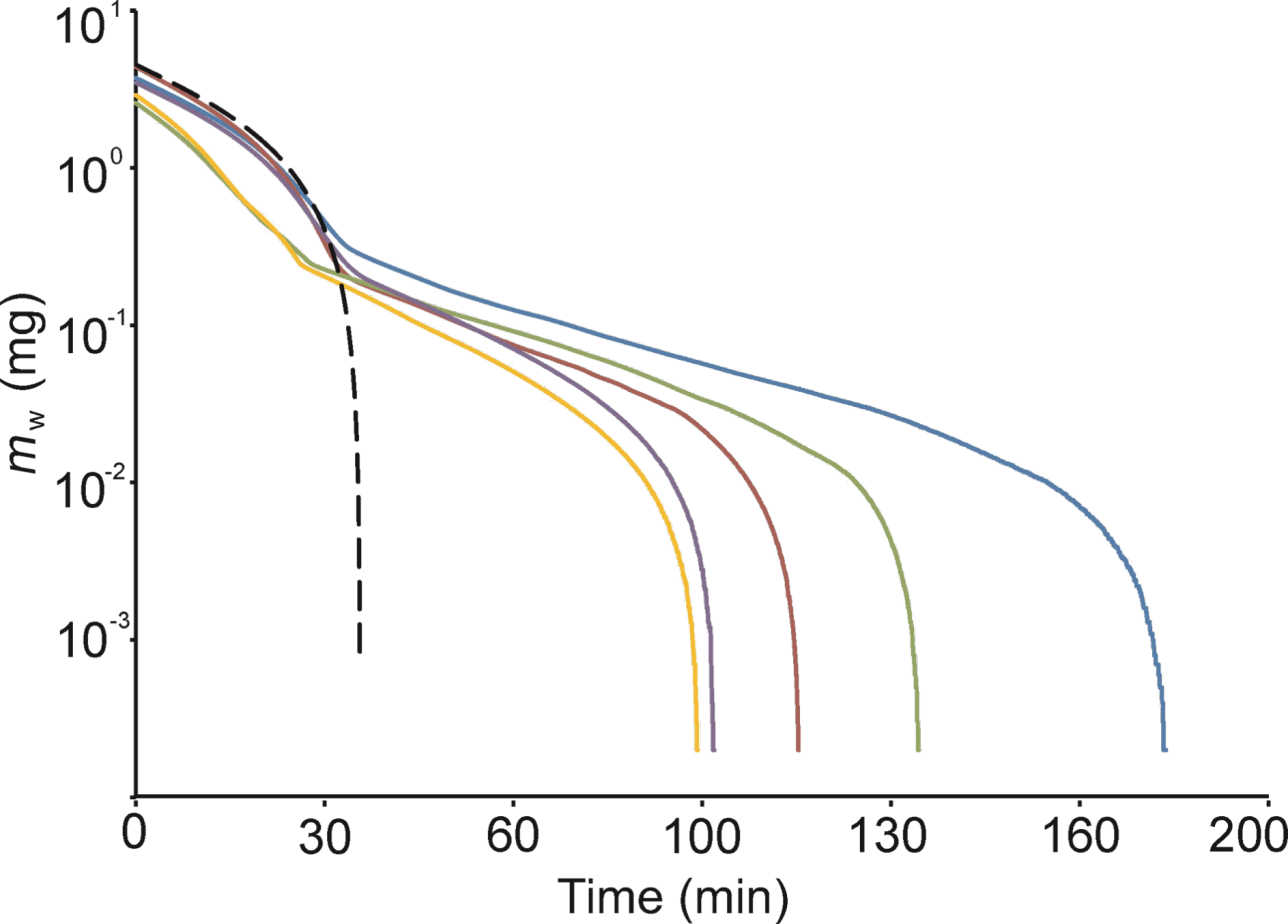
Desiccation dynamics of five individual plantain seeds (solid lines) and a water droplet (dashed line). *m*_w_ is mass of absorbed water or a water droplet mass. The first 30 min of desiccation of the water droplet and mucilage are comparable. After about 35–40 min the mucilage lost about 91% of the water while the water droplet completely evaporated.

The dry seed mass varied slightly between individual seeds (Table 1). After hydration, the maximum mass increased approximately fourfold, but differed slightly between the seeds (Table 1). The mass of absorbed water differed from 2.5 mg to 4.3 mg. The drying time (elapsed time until the fully hydrated seed achieved its dry mass) ranged from 90 to 180 min (Figure 2). The desiccation dynamics of the seeds clearly show three distinguishable phases: 30 min after hydration, 45 min after hydration and the latest linear phase. At a time of 30 min after hydration, the seeds lost 87.3 ± 1.7 % (mean ± SE) of the absorbed water, similar to the evaporation behavior of a water droplet (Figure 2). The nonlinear desiccation dynamics is easy to see in Figure 2. The deviation from linear dynamics may be related to the decreasing area from which water evaporates and a change in physicochemical properties of the mucilage depending on the water concentration. In two seeds the first phase was shortened to 15 min with followed by two sudden drops in the evaporation rate (Figure 2). These drops should correspond to jumps in the evaporation area. The second desiccation phase starts around 40 min after hydration. It is characterized by exponential desiccation and lasts for between 20 to 100 min. Such dynamics are typical for a physicochemical equilibrium and may be related to the evaporation of water bound to pectin molecules. The final desiccation phase is again linear and could be related to the slow evaporation of the rest of the water without change in the evaporation area. The rate of water loss during the first 35–40 min is comparable to the evaporation rate of a water drop.

**Table 1:** The mass of *Plantago lanceolata* seeds before and after hydration.

seed	seed dry mass (mg)	seed mass after hydration (mg)	mass of absorbed water (mg)	seed mass increase after hydration (%)

1	1.3	5.7	4.3	410
2	1.5	4.1	2.5	260
3	1.7	5.2	3.4	290
4	1.3	4.2	2.9	310
5	1.3	5.0	3.7	380
average ± SD	1.4 ± 0.1	4.8 ± 0.6	3.4 ± 0.6	330 ± 62.8

### Adhesive forces and contact area

**Figure 3:**
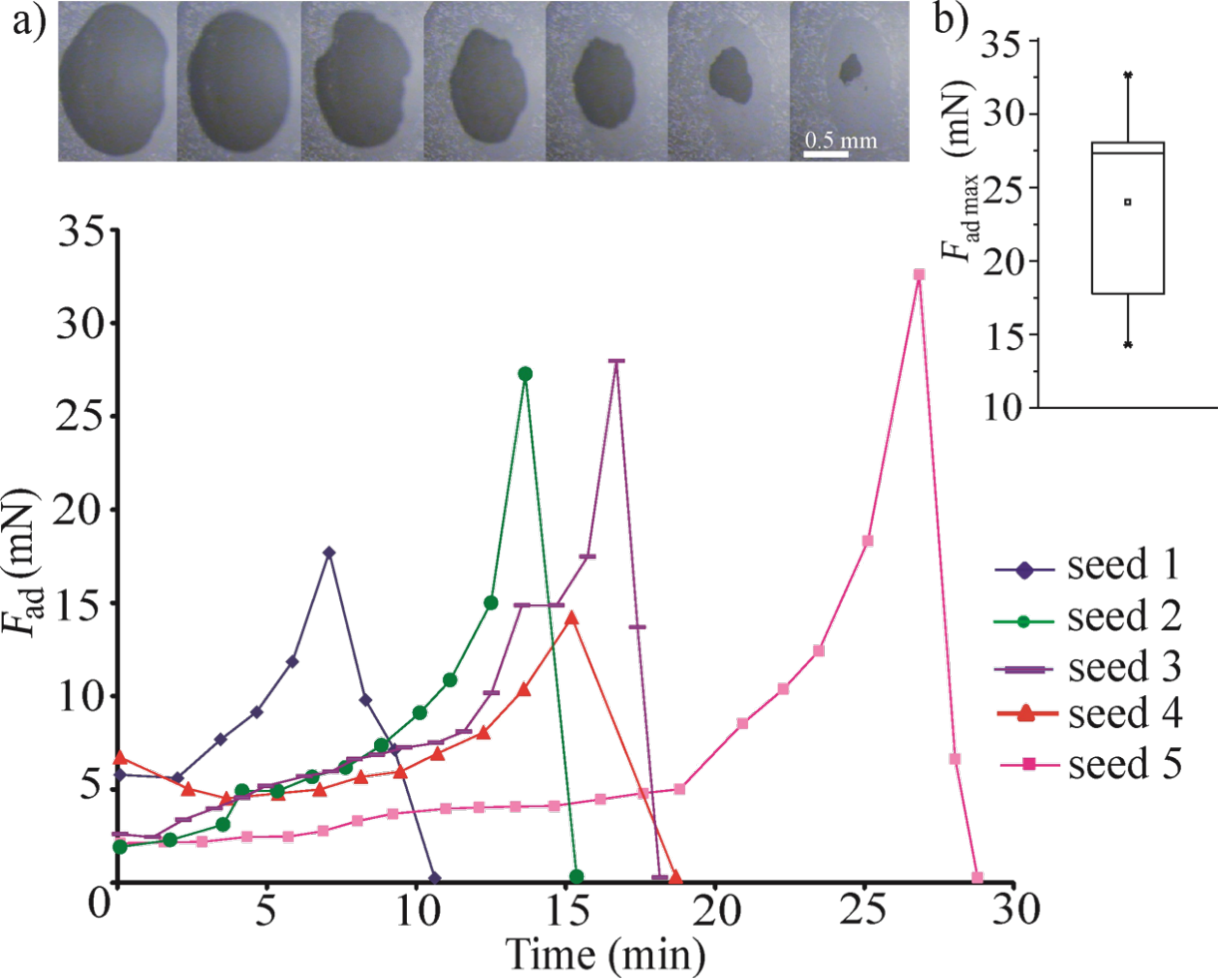
Adhesion force (*F*_ad_) of mucilaginous seeds measured at different degrees of desiccation after full hydration. (a) Dynamics of the adhesion force is shown for five individual seeds. The top inset of microscopic images illustrates the decreasing contact area of one individual seed with the glass block under load during pull-off measurements. Interestingly, the adhesion–time curves of the individual seeds are comparable; (b) Box-and-whisker diagram of the mean maximum adhesion force of measured seeds, where the bottom and top of the box are the 25th and 75th percentiles, the band inside the box is the median; the ends of the whiskers are the 10th and 90th percentiles.

**Figure 4:**
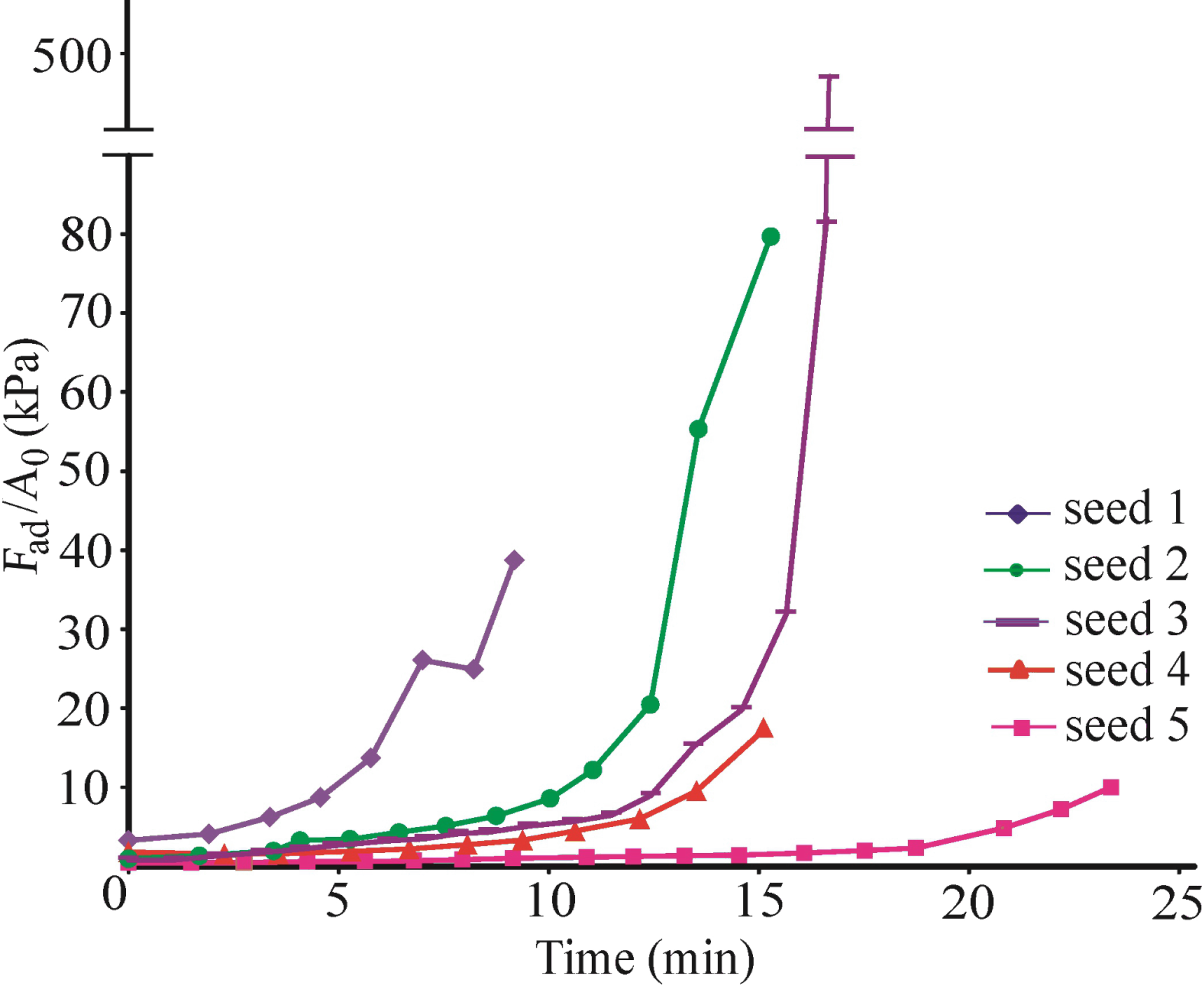
The ratio of pull-off force (*F*_ad_) to contact area (*A*_0_) (adhesion stress) for five individual seeds as a function of time. The smallest adhesion stress was observed for the seed demonstrated the highest contact area and pull-off force.

The pull-off force of individual *P. lanceolata* mucilaginous seeds varied up to two orders of magnitude, 0.3–32 mN (Figure 3). Immediately after hydration, the pull-off force was low whereas the contact area between the mucilage and substrate was the largest. The pull-off force increased and reached a maximum when the mucilage envelope lost about 45–50% of water. The maximal pull-off force of individual seeds ranged between 14 and 32 mN (mean value 24 ± 3 mN). The contact area of fully hydrated seeds varied from 1.8 to 5 mm^2^ and was below 0.01 mm^2^ when the entire amount of absorbed water had been evaporated. At the same time, the adhesion stress, *F*_ad_/*A*_0_, continuously increased with time (Figure 4). The maximum adhesion stress reached 480 kPa. The seeds demonstrated detectable adhesion until between 11 and 29 minutes after hydration (Figure 3), presumably because of a different mucilage amount and water content in the envelope. The average time for reaching the maximum force was 16 ± 3 min. Very important and interesting was that the shape of the time-dependent pull-off force curves of individual seeds are very similar (Figure 3). After reaching a maximum value, they decreased rapidly and no adhesion was further detected.

### Friction properties

**Figure 5:**
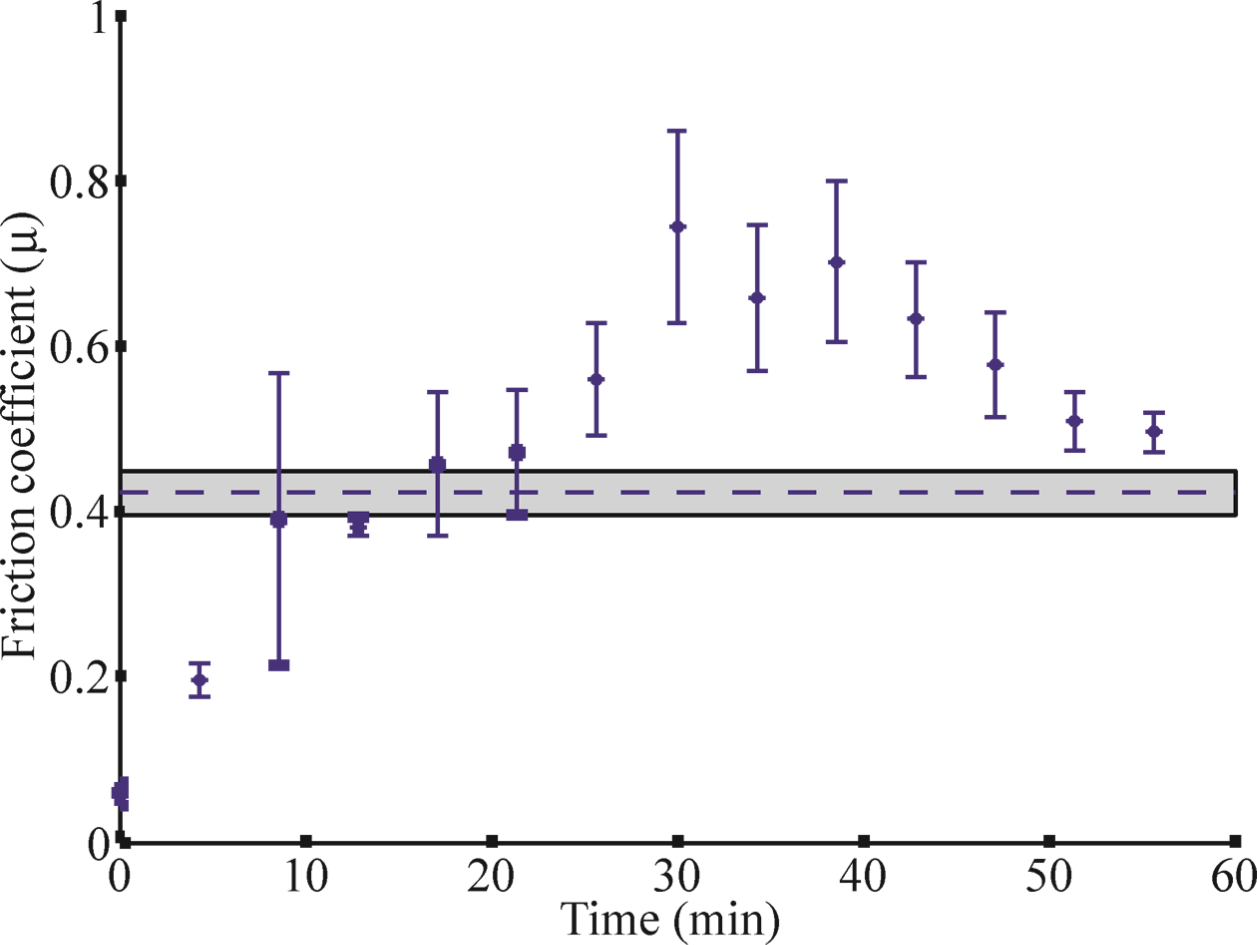
Dynamics of the friction coefficient, µ. The friction coefficient was calculated according to Equation 2 with λ = 7.75 and *c* = 0.012. The mean value of the friction coefficient for dry seeds is shown by the dotted line inside the gray box (standard deviation). Mean values of the friction coefficient for hydrated seeds are shown by stars, and error bars indicate standard deviation.

The mean values of the friction coefficient, obtained for dry seeds of *P. lanceolata*, ranged from 0.4 to 0.5 (mean value 0.4 ± 0.01). Freshly hydrated seeds had an extremely low friction coefficient of about 0.05–0.08 (mean value 0.06 ± 0.01). Subsequently, it rapidly increased during desiccation and, after 30 min of desiccation time, reached values of around 0.5–0.8 (mean value 0.74 ± 0.06) (Figure 5). At the same time, the mucilage envelope lost almost the entire amount of absorbed water. The highest measured value of the friction coefficient was 0.8. Because of the strong adhesion this friction coefficient was observed at a platform declination of 73°. After the maximum was reached, the friction coefficient slowly decreased (Figure 5). When the seeds had completely dried out (after about one hour after the hydration procedure), the values of the friction coefficient were comparable to those of the dry seeds. Interestingly, the maximal adhesion was reached earlier (at average desiccation times of about 16 min, see above).

## Discussion

We demonstrated that the seed mucilage of *Plantago lanceolata* represents a type of cellulose mucilage, which differs clearly in the physical properties from the pectic mucilage of previously studied *Linum usitatissimum* [32]. Because of the presence of cellulose, the mucilage of *P. lanceolata* represents a more ordered and stable system in comparison to the pectic type. This correlates with a weaker adhesion of this type of mucilage.

### Cellulose fibrils are important for the distribution and retaining of the mucilage on the seed surface

The *P. lanceolata* mucilage was classified as “true slime”, consisting of pectins as the main component [12]. However, our study revealed, for the first time for this taxon, the presence of an additional, important component of the mucilaginous envelope, namely the cellulose fibrils. Specific staining reactions visualized delicate cellulose fibrils stretching out from the seed surface into the pectin mass and forming a regular, radially arranged skeleton. This kind of mucilage with cellulose fibrils is characteristic of many taxa from such families, as Asteraceae, Brassicaceae, Lamiaceae [1,13,15–16,34]. Also, in other *Plantago* species (*P. media, P. ovata*), we detected the presence of cellulose fibrils [14,37].

### Water loss from the mucilage envelope

The maximum mass gain of *Plantago lanceolata* mucilaginous seeds after hydration was fourfold (4 mg) (Table 1) whereas in *Linum usitatissimum,* having a pectic envelope and lacking a cellulose “skeleton”, it was a maximum of threefold (15 mg) [32], (t-test, *p* = 0.04). However, the water loss in the mucilage of *P. lanceolata* was much more rapid. After about 35 min., *P. lanceolata* mucilage lost, on average, 92% of absorbed water (Table 2), whereas flax seed mucilage at the same time lost, on average, only about 56% of absorbed water (t-test, *p* = 0.003).

**Table 2:** Comparison of desiccation data for *Plantago lanceolata* (cellulose mucilage) and *Linum usitatissimum* (pectic mucilage).

measured feature	*Plantago lanceolata* mean ± SD (min–max)	*Linum usitatissimum* mean ± SD (min–max) [32]

mean water loss after 35 min, %	92 ± 0.8 (90–94)	57 ± 8 (37–78)
mass of absorbed water, mg	3 ± 0.3 (3–4)	10 ± 2 (6–15)
desiccation time, min	128 ± 15 (99–182)	229 ± 9 (206–252)

The explanation of the differences in water absorption and desiccation between pectic and cellulose mucilage can be the chemical factors that influence the pectins structure and properties. For example a higher degree of methylation increases the capacity to form gels, whereas gelling is inhibited by increasing acetylation [38–39]. The presence of calcium ions slows down water transfer to cellulose and pectins and thus increases the cell-wall stiffness [19,40–41]. Also the presence of boron in the pectic network causes the reduction of the amount of water and contributes the cell-wall strength [19,40]. We can surmise that the existence of such interactions within the pectin–cellulose network can cause changes in the spatial structure of the mucilage and affects the hydration and dehydration processes.

The cellulose mucilage composition and structure can be also a reason for observed two main evaporation phases. The mucilaginous envelope shows two regions, an outer region composed mostly of unbranched pectins without cellulose and an inner region with cellulose fibrils and branched pectins [42–43]. The presence of branches is responsible for the formation of bonds with other molecules [19]. The components could form a mesh-filled network entrapping water for longer time [44]. This could be the reason of faster water evaporation from the outer layer (first phase) and slower (second phase) from the inner part of the envelope.

### Mucilage adhesive properties

The mucilage adhesion increases with increasing water loss from the mucilage envelope [32]. In case of cellulose mucilage of *P. lanceolata,* the maximum adhesion was reached earlier in comparison to the pectic mucilage of *L. usitatissimum*. The possible reason for this behavior can be a higher desiccation rate of *P. lanceolata* mucilage and a special mucilage structure (mentioned above). This can also cause a faster fixation of *P. lanceolata* seed to the surface of, e.g., the body of an animal.

**Figure 6:**
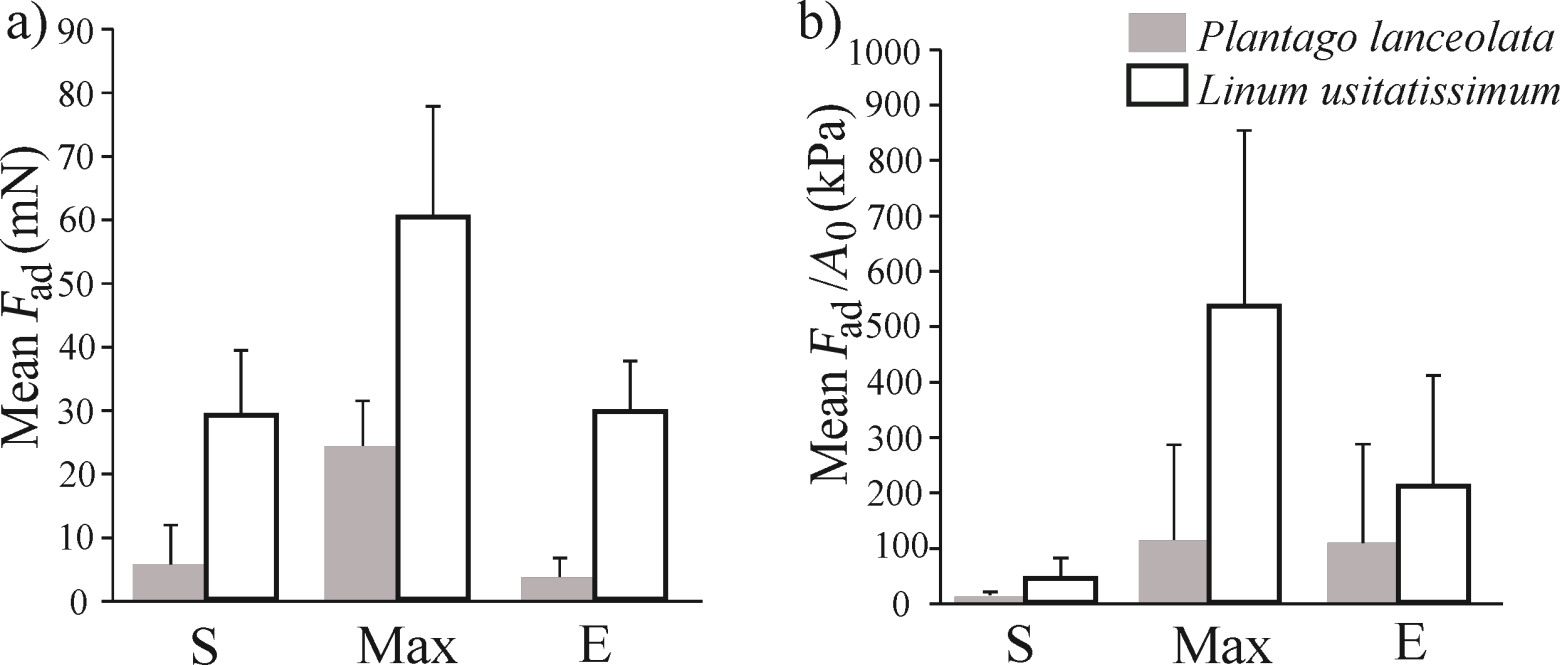
The comparison of the mean values of *F*_ad_ (adhesion force) and *F*_ad_/*A*_0_ (adhesion stress) at different desiccation conditions for *Plantago lanceolata* and *Linum usitatissimum*. S – at the beginning of the experiment, when the mucilage envelope contains water, Max – maximal value (when the mucilage envelope probably lost the most amount of water), E – at the end of the measurements (when the mucilage almost dried out).

The *P. lanceolata* mucilage had a statistically significant smaller maximum pull-off force than the pectic mucilage of flax [32] (Figure 6a, t-test, *p* = 0.002). Our measurements of maximum adhesive forces demonstrated 2.5 times lower values for *P. lanceolata* cellulose mucilage in comparison to the pectic type [32] (t-test, *p* = 0.008). The maximal value for plantain seeds was just 33 mN whereas for flax it was 91 mN (Figure 6a, Table 3). The time for reaching maximum adhesion was half as long as for plantain (16 min) than for flax (30 min) (Table 3). Furthermore, the adhesion stress values at pull-off (*F*_ad_/*A*_0_) were 4.5 times smaller for plantain in comparison to flax (Figure 6b) (t-test, *p* = 0.04).

**Table 3:** Comparison of adhesion force data for *Plantago lanceolata* (cellulose mucilage) and *Linum usitatissimum* (pectic mucilage).

measured feature	*Plantago lanceolata* mean ± SD (min–max)	*Linum usitatissimum* mean ± SE (min–max) [32]

maximum adhesion force *F*_a_, mN	24 ± 3 (14–33)	60 ± 8 (45–92)
contact area at *F*_a_ (*A*_0_), mm^2^	0.6 ± 0.1 (0.3–1)	1.2 ± 0.6 (0.2–3.5)
mean time for reaching *F*_a_, min	16 ± 3 (7–27)	29 ± 5 (15–45)

### Adhesive properties of the cellulose mucilage

We infer that both chemical composition and structure of the mucilage plays an important role in adhesion. In the cell wall of MSCs, cellulose, hemicelluloses and pectins can be linked covalently in a form of network structure. Such an association between these polysaccharides has been described for typical cell wall, as well as for the wall of MSCs [17,41]. In *P. lanceolata,* pectins could be attached to the cellulose fibrils leading to a decrease in adhesive force as, most likely, not all pectin side chains can be involved in the adhesion processes on the surface. The cellulose microfibrils also provide mechanical stability for the cell wall [45]. This fact can explain the regular and comparable shapes of the adhesion–time curves obtained for individual *P. lanceolata* seeds. They looked very similar in their character. In the case of *L. usitatissimum*, mucilage viscosity strongly depends on the mixture of RG I (rhamnogalacturonan I) and arabinoxylan [42]. In *L. usitatissimum,* the adhesion–time curves of individual seeds differed strongly [32]. It can confirm that the presence of the cellulose in the mucilage of *P. lanceolata* acts as a stabilizing factor.

### Mucilage adhesive properties in epizoochoric seed dispersal

Adhesion, by the mucilage, to feet, feathers or fur of animals, is a very effective dispersal method. Many different seeds are transported in this way between remote islands, including seeds of some *Plantago* species [8,46]. The ability of the seeds to adhere appeared shortly after hydration and became stronger with desiccation resulting in a strong sticking to an animal as dispersal agent. At this stage, the seeds can strongly adhere to, e.g., the plumage of birds. After drying, they can be actively or passively removed again.

In the case of flax mucilage, the adhesion ability intensified and degraded gradually whereas in plantain the adhesion ability, after reaching a maximum, degraded rapidly. The enhanced adhesion stress and quicker development of the maximal adhesion should allow the seed with cellulose mucilage (*P. lanceolata*) to attach more quickly to the surface. Also, the dynamics of water evaporation can be important for mucilaginous seed fixation to the ground or to the bodies of animals. The adhesion to bird plumage of diverse *Plantago* taxa mucilaginous seeds was also observed, providing an example of epizoochory for this species [8]. *P. lanceolata* counts to the wide spread species over the whole North America and to one of the world’s 12 successful, non-cultivated colonizing species occurring almost throughout the whole world, what demonstrates a great potential of mucilaginous diaspores as dispersal units [5,7].

### Frictional properties of mucilage and their role in endozoochoric seed dispersal

For freshly-hydrated plantain seeds, we observed low values of friction coefficient (0.06 ± 0.007; 0.05–0.08). It was slightly higher than those previously observed for hydrated flax seeds (0.05 ± 0.01; 0.04–0.05) [25] (Table 4, t-test, *p* < 0.001). The mucilage of plantain represents a heterogenous system composed of pectins and cellulose fibrils. Therefore, the mucilage of *P. lanceolata* exhibited a higher friction than the pectic mucilage of *L. usitatissimum*. However, the friction of *P. lanceolata* mucilaginous seeds should be sufficient for endozoochoric dispersal. *P. lanceolata* seeds can be eaten by birds, and because of the mucilage, which provides lubrication, the seeds can pass easier through the digestive system of the birds to be spread via this means. Viable seeds of plantain were found in pigeons, sparrows, bullfinch, greenfinch and cattle droppings [5,8]. It was also observed that seeds of plantain, which usually have 56% germination, yielded 100% germination after passing through a bird’s digestive system [5]. Such examples of dispersal of mucilaginous seeds were observed in nature, e.g., for *Cecropia* [3].

**Table 4:** Comparison of friction data for *Plantago lanceolata* (cellulose mucilage) and *Linum usitatissimum* (pectic mucilage).

measured features	*Plantago lanceolata* mean ± SD (min–max)	*Linum usitatissimum* mean ± SD (min–max) [32]

friction coefficient, µ, of dry seeds	0.4 ± 0.01 (0.4–0.4)	0.2–0.3
friction coefficient, µ, of freshly hydrated seeds	0.06 ± 0.007 (0.05–0.08)	0.045 ± 0.01 (0.04–0.05)
Max sliding angle	73°	180°

## Conclusion

In this study, we demonstrated structural and physical properties of mucilaginous seeds of *Plantago lanceolata*. The seed mucilage of *P. lanceolata* is characterized by the presence of cellulose fibrils. The loss of water from the mucilage envelope is a very dynamic process, affecting both the adhesive and frictional properties of the mucilage. Maximal adhesion was measured for mucilage after some degree of water loss. The lowest friction was measured in fully hydrated seeds. The presence of cellulose fibrils in the mucilage envelope can be responsible for the faster desiccation and weaker adhesion of plantain seed mucilage compared to flax. Since the cellulose mucilage *P. lanceolata* represents a more regularly ordered system than the pectic mucilage of *L. usitatissimum*, the course of adhesion force over desiccation time was more regular in plantain seeds when compared to flax. The presence of cellulose also results in the decrease of seed friction in its fully hydrated condition. Cellulose mucilage demonstrated weaker adhesive and frictional properties than the pectic type but it still indicates an effective system promoting zoochoric dispersal and/or attachment to the substratum (soil). As the *P. lanceolata* seed mucilage represents a special type of the cell wall rich in pectins, which is loosely organized and easy accessible, this type of mucilage could also be considered as a model for further mechanical studies of such modified type of the cell wall.
